# Is There a Burnout Epidemic among Medical Students? Results from a Systematic Review

**DOI:** 10.3390/medicina60040575

**Published:** 2024-03-30

**Authors:** Matteo Di Vincenzo, Eleonora Arsenio, Bianca Della Rocca, Anna Rosa, Lucia Tretola, Rita Toricco, Alessia Boiano, Pierluigi Catapano, Sandra Cavaliere, Antonio Volpicelli, Gaia Sampogna, Andrea Fiorillo

**Affiliations:** Department of Psychiatry, University of Campania “Luigi Vanvitelli”, 80138 Naples, Italysandracavaliere.na@gmail.com (S.C.);

**Keywords:** medical students, burnout, prevention, severe mental disorder, mental exhaustion

## Abstract

*Background and Objectives*: Medical students represent the ideal target group for promoting mental health and mental wellbeing, being exposed to specific risk factors, such as the content of medical training, the exposure to sickness and death, and a stressful academic routine. Medical students report high levels of cynicism and emotional exhaustion, which represent two of the essential features of burnout syndrome. In this systematic review, studies assessing the levels of burnout among medical students through validated tools worldwide were analyzed. *Materials and Methods*: A systematic review has been performed in order to identify studies: (1) focusing on samples of medical students; (2) evaluating burnout syndrome using validated tools; (3) providing prevalence data on burnout; and (4) written in English. *Results*: Out of the 5547 papers initially obtained, 64 were finally included in the analysis. The sample sizes ranged from 51 to 2682 participants. Almost all studies had a cross-sectional design; the Maslach Burnout Inventory and its related versions were the most frequently used assessment tools. The prevalence of burnout, which was stratified based on gender and academic stage, ranged from 5.6 to 88%. Burnout was mostly predicted by thoughts of stopping medical education, negative life events, lack of support, dissatisfaction, and poor motivation. *Conclusions*: The prevalence of burnout syndrome in medical students is quite heterogeneous, reaching a peak of 88% in some countries. However, several predictors have been identified, including negative life events or poor motivation. These findings highlight the need to develop preventive interventions targeting the future generation of medical doctors, in order to improve their coping strategies and resilience styles.

## 1. Introduction

In recent years, the need to promote mental health and wellbeing among young people has been repeatedly affirmed [1,2]. Youth represents a crucial developmental phase, and many mental disorders have their onset in adolescence and early adulthood. A longitudinal study showed that 73.9% of adults with a mental disorder received a diagnosis before 18 years of age and 50.0% before 15 years of age. Mental disorders may, in part, be triggered by stress exposure in adolescence and early adulthood. The exposure to any biological, social, or psychological risk factor during early adulthood can have a disproportionate impact. Therefore, several strategies have been proposed for promoting mental wellbeing and mental health, mainly including the adoption of healthy lifestyles [1].

Among young people, the group of medical students is at a particular high risk of developing mental disorders [3]. In fact, common mental disorders, including anxiety and depressive disorders, range between 27% and 34% among medical students [4], being significantly higher in medical students when compared to non-medical peers of the same age. The data are alarming when looking at the levels of suicidal ideation in medical students, reaching a peak of 11% [5,6,7]. These findings might be due to the peculiar features of a medical school career. In fact, the organization of the training program at medical school is very complex, including many examinations, lectures, and practical activities. Therefore, medical students are exposed to a significant amount of organizational stress due to the many commitments to fulfill. Moreover, medical students deal with the continuous exposure to sickness and death, which requires both technical and personal skills (such as coping strategies and resilience). Medical students might be very competitive, especially when specific personality traits, mental rigidity, the fear of failure, and the desire for perfectionism are present [8]. Furthermore, the high levels of cynicism and emotional exhaustion among medical students also contribute to the occurrence of psychiatric symptoms and of burnout syndrome [9,10,11,12,13,14]. Medical students tend to, consequently, hold themselves to a high standard to succeed academically. The work-related and financial stressors drive students to pursue competitive specialties; this performance pressure is further exacerbated during the clinical years, where students are not only expected to succeed on their shelf exams but are also being constantly evaluated by their preceptors and residents. Being under constant scrutiny creates an enormous amount of stress, particularly during the student’s rotation on their specialty of choice, where they would like to secure strong letters of recommendations. After a long day in the wards where students feel scrutinized, they are expected to go home and spend their evening studying and reading for their exams.

Burnout is defined as a syndrome “resulting from chronic workplace stress that has not been successfully managed” [15]. The term was first introduced in the psychological research by Freudenberger in 1974 [16] in order to describe the depletion of the energy and motivation of healthcare professionals. Burnout is characterized by three core dimensions [17]: emotional exhaustion; feelings of depersonalization, negativism, or cynicism; and reduced personal accomplishment. Emotional exhaustion reflects the levels of stress, and, although it is a necessary criterion, it is not sufficient by itself to define burnout syndrome. Depersonalization is the attempt to react to exhaustion by taking a cognitive distance from the job, while reduced personal accomplishment indicates how professional effectiveness may be affected by the other two dimensions.

Burnout research was originally confined within the frame of human services and healthcare professionals [17]. Since 1990s, this syndrome began to be investigated among other professional groups, such as managers, military, and also medical students [18,19]. In the last decades, new instruments were introduced for evaluating burnout levels regardless of the three-dimension model proposed by Maslach et al. [17]. This complexity contributed toward generating heterogeneity in the prevalence rates of burnout syndrome.

Given their exposure to high levels of stress and study-related pressure, medical students are particularly prone to developing burnout. A meta-analysis examining 24 international studies found that almost one out of two medical students before residency suffer from burnout, with prevalence rates varying according to geographic area [20]. Common stressors for medical students include the examination load, sense of expectation, competition with peers, loss of family contacts, repeated rotations, lack of team spirit, poor sleep hygiene and diet habits, and financial and/or logistic issues [21,22,23]. Nevertheless, medical students have not received the same attention paid to medical doctors in terms of burnout research [24].

Although burnout is not yet considered a proper mental disorder, it often turns into proper mental disorders, including insomnia, and depressive and anxiety disorders. Therefore, the assessment of levels of this syndrome among medical students could represent a proper way to develop prevention strategies and to implement adequate support strategies within medical schools.

Based on these premises, the present systematic review has been promoted in order to: (1) provide updated information on the prevalence rates of burnout syndrome in medical students worldwide; (2) to identify assessment measures most commonly used in clinical practice; and (3) to identify predictors and protective factors for burnout syndrome in medical students.

## 2. Materials and Methods

This systematic review has been carried out according to a multi-step procedure. The following steps were performed: (1) definition of the problem; (2) search in the literature; (3) evaluation of data; (4) analysis of data; and (5) presentation of results. Prevalence of burnout in medical students of different age, gender, grade, and cultural background, as well as assessment tools used to evaluate burnout, were considered as outcomes of interest. Predictors and protective factors were reported when available.

**Figure 1:**
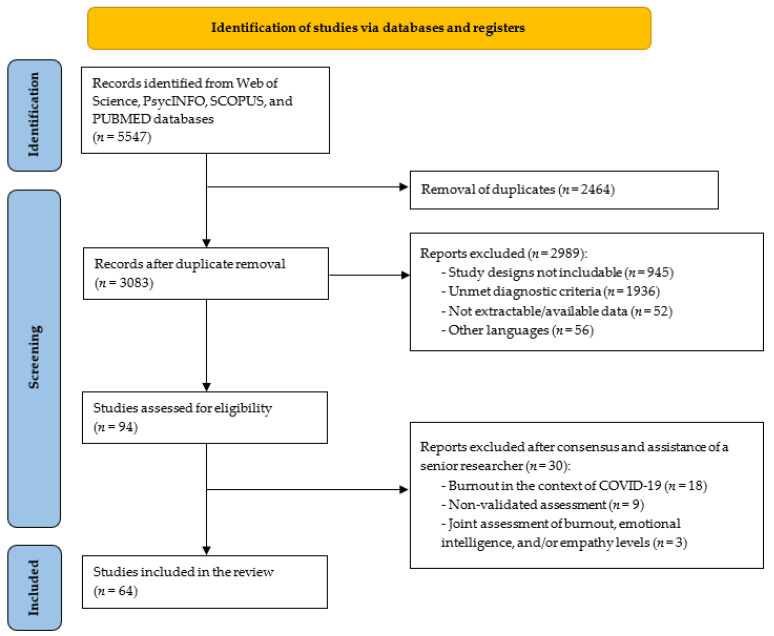
PRISMA flowchart for study selection.

The search terms “med* student* AND med* school OR universit* AND burnout” were entered into Web of Science, PsycINFO, SCOPUS, and PubMed. The Boolean search technique, which consists of a logical information retrieval system, was applied by combining two or more terms to make searches more restrictive or detailed. Databases were searched from the inception of each source to 31 October 2023. The review process was performed in accordance with PRISMA guidelines and a PRISMA flowchart has been included (Figure 1). Studies were finally considered if they: (a) included samples of medical students; (b) used validated tools to assess burnout (i.e., tools whose accuracy and reliability have been confirmed); (c) provided prevalence data on burnout; and (d) were written in English. Studies were excluded if they: (a) included students from the medical and non-medical fields, without providing sub-group data; (b) assessed burnout of medical students during the COVID-19 pandemic; or (c) were reviews, meta-analyses, study protocols, case reports, comments, letters to editor, expert opinions, or qualitative studies.

### 2.1. Study Selection and Data Extraction

Search of literature, title–abstract screening, and full-text screening were performed by independent researchers (MDV, AB, EA, LT, SC, RT, and AR). References of relevant articles were hand-searched to evaluate further papers of potential interest. The search process was carried out with a double-blind methodology; discrepancies were solved through consensus, and a senior researcher (GS) was consulted, if needed. For each paper, the following information were collected by using an ad hoc extraction tool: authors, country and year of publication, sample size, burnout prevalence, assessment tools, dimensions of burnout, predictors of burnout, and protective factors. Authors screened the articles identified by the searches, and then performed a full-text review of those selected by titles and abstracts. Disagreements that arose between the reviewers were resolved through discussion and, in the case of continued disagreement, with the assistance of a senior researcher (GS). AF reviewed the full study methodology and provided comments to improve papers’ search and extraction.

### 2.2. Risk of Bias

As suggested by the Cochrane review methodology, for each included study, the risk of bias (RoB) was assessed by using the Risk Of Bias In Non-randomized Studies of Interventions (ROBINS-I) tool [25], which provides a framework for assessing the risk of bias in a single result (an estimate of the effect of an experimental intervention compared with a comparator intervention on a particular outcome). Two researchers with proven experience in the field of risk of bias assessment (AB and EA) evaluated the studies. Discrepancies were solved through discussion with a senior researcher (GS), if needed.

The overall risk of bias was rated as high; Supplementary Table S1 shows the results of risk of bias assessment.

## 3. Results

A total of 5547 records were initially obtained. After removing 2464 duplicates, 3083 papers were screened and *n* = 2989 records were excluded due to the study design (*n* = 945), unclear diagnostic criteria (*n* = 1936), data unavailability (*n* = 52), and non-English language (*n* = 56). Ninety-four full-text papers were examined, and only 64 studies were included in the analysis. Of the 30 excluded studies, *n* = 18 studies explored burnout during the COVID-19 pandemic, *n* = 9 studies assessed burnout with non-validated tools, and three studies did not separate the data on burnout from those on the levels of empathy and emotional intelligence. The main findings of the included papers are reported in Table 1.

According to the evaluation of the risk of bias, the majority of studies were scored with a high risk (*n* = 31, 48.4%), while only nine papers reported a low risk of bias (14.1%). All the details are reported in Table S1.

The first paper to be included in our review was published in 2006 [26], while four papers were published in 2023 [27,28,29,30]. The samples of students included in the studies ranged from 51 [31] to 26,123 [32]. Almost all studies (62 out of 64 included) [26,27,28,29,30,31,32,33,34,35,36,37,38,39,40,41,42,43,44,45,46,47,48,49,50,51,52,53,54,55,56,57,58,59,60,61,62,63,64,65,66,67,68,69,70,71,72,73,74,75,76,77,78,79,80,81,82,83,84,85,86,87] were cross-sectional, while longitudinal analyses were performed by Cortez et al. [88] and by Thun-Hohenstein et al. [89] only.

**Table 1:** The main findings of included papers.

	Authors, Country (Year)	Sample	Burnout Prevalence	Assessment Tools	Prevalent Dimension	Positively Associated Variables and Predictors of Burnout	Protective Factors
1	Dyrbye et al., USA (2006) [26]	*n* = 545 (M: *n* = 247; F: *n* = 297)	45%	MBI	EE: low (37.5%); moderate (27.8%); high (34.7%). D: low (52%); moderate (22.4%); high (25.8%). PA: high (42%); moderate (27.2%); low (30.8%).	Negative life events experienced in the previous 12 months.	Positive life events in the previous 12 months.
2	Dyrbye et al., USA (2007) [33]	*n* = 1701 (M: *n* = 777; F: *n* = 918; Not specified: *n* = 6) Ethnic minority: *n* = 410	47%	MBI	High EE: 600/1603 (37%); High D: 435/1555 (28%); Low PA: 413/1480 (28%).	Non-minority students (non-racially/ethnically diverse students) were more likely to be burnt-out and have higher EE and DP scores. Burnout was associated with racial discrimination and prejudices, isolation, and different cultural expectations among minority students.	
3	Dyrbye et al., USA (2010) [34]	*n* = 2682 (M: *n* = 1352, 51.4%; F: *n* = 1280, 48.6%; Missing: *n* = 50)	52.8%	MBI	EE: 24.4 (10.59%); D: 8.0 (5.92%); PA: 36.0 (7.31%).	Having engaged in cheating/dishonest/unprofessional clinical behaviors; less altruistic views regarding physicians’ responsibility to society; less consistent opinions regarding appropriate relationships with industry.	
4	Galán et al., Spain (2011) [35]	*N* = 447 (M: *n* = 130; F: *n* = 317)	22.6%	MBI-SS	EE: 17.8%; CY: 10%; LOW PE: 17.8%.		
5	Reed et al., USA (2011) [36]	*n* = 1192 (M; *n* = 53.9%; F: *n* = 46.1%)	45.6%	MBI SF-8	EE: 24.4 (±10.5); D: 5.6 (±5.3); PA: 34.6 (±8.3).	Compared with school students using pass/fail grading, using grading scales with 3 or more categories was associated with higher stress, burnout, EE, and D.	To spend a greater percentage of their contact hours in clinical experiences.
6	Chang et al., USA (2012) [37]	*n* = 526	55%	MBI-HSS PRIME-MD PMSSS	EE: 51.7%; D: 44.0%; PA: 52.0%.		Social support; counseling services; extracurricular activities.
7	Cecil et al., UK (2014) [38]	*n* = 356 (M: *n* = 124; F: *n* = 232)	26.7%	MBI	EE: 55%; D: 34%; LOW PA: 46.6%.	EE: Physical activity, smoking. D: Male gender, year of study, and institution. PA: Alcohol binge score, year of study, gender, and physical activity.	
8	Cook et al., USA (2014) [39]	*n* = 564 (M: *n* = 306; F: *n* = 258) Medical students undergoing recurrent mistreatment *n* = 59	Overall: 34.1% Recurrent mistreatment by faculty: 57.4% Recurrent mistreatment by residents: 49.1%	MBI-2		Recurrent mistreatment (several times) by faculty or residents.	
9	Dyrbye et al., USA (2014) [40]	*n* = 4402 (M: *n* = 1972; F: *n* = 2404)	55.9%	MBI	EE: 44.6%; D: 37.9%; LOW PA: 31.3%.	Low sense of accomplishment.	
10	Dyrbye et al., USA (2015) [41]	*n* = 873 (M: *n* = 442 (50.9%); F: *n* = 431 (49.1%))	52.7%	MBI SF-8			
11	Seo et al., Korea (2015) [42]	*n* = 263 (M: *n* = 141; F: *n* = 122)	9.9%	MBI	EE: 28.1%; D: 40.4%; LOW PA: 31.3%.	Educational stressors and relational stressors; female gender, second school year, Catholic religion, bad self-rated health.	Social support.
12	Almeida et al., Brazil (2016) [43]	*n* = 376	14.9% M = 15.2 (23%) F = 15.3 (33%)	MBI-HSS		Having failed examinations; having considered abandoning the course.	
13	Asencio-Lòpez et al., Mèxico (2016) [44]	*n* = 225 Year 1–3: 153 Year 4–6: 72	Year 1–3: 2.8% Year 4–6: 8.3%	MBI			
14	Thompson et al., USA (2016) [45]	*n* = 161 (M: 46.6%; F: 53.4%)	48.5%	MBI	Low EE: 58/140 (41); Average EE: 35/140 (25); High EE: 47/140 (34). Low PA: 36/134 (27); Average PA: 36/134 (27); High PA: 62/134 (46). Low D: 50/133 (38); Average D: 39/133 (29); High D: 44/133 (33).	Thinking of not receiving support from family and friends is associated with high EE; higher likelihood of having low PA in those who do not think to receive help and support from other medical students compared to those who did.	
15	Youssef et al., Republic of Trinidad and Tobago (2016) [46]	*n* = 381 (F: 67%) Years 1–3: 59%	53%	MBI	EE: 27.1 ± 11.4; D: 8.1 ± 6.6; PA: 31.3 ± 8.3.	EE and D scores were significantly higher in year 5.	Time for relaxation, emotional support.
16	Bughi et al., USA (2017) [47]	*n* = 185	45.4%	MBI GWB MBI-SS	High EX: 118/182 (64.8%); High CY: 76/182 (41.8%); Low PE: 38/182 (20.9%).		
17	Popa-Velea et al., Romania (2017) [48]	*n* = 299 (M: *n* = 94; F: *n* = 205)	15.05%	MBI	EE: 16.72%; D: 28.42%; Low PA: 10.7%.	Perceived stress, alexithymia, low perceived social support.	
18	Ali et al., Pakistan (2018) [49]	*n* = 373 (M = 120; F = 253)	61.1%	OLBI	DI: 56.3%; EX: 58.7%.	DI: pre-clinical years. EX: female gender, clinical years.	
19	Barbosa et al., Brazil (2018) [50]	*n* = 399 (M = 177; F = 222)	12%	MBI-SS	EE: 63.2%; D: 44.6%; LOW PE: 22.6%.	Female gender.	
20	Boni et al., Brazil (2018) [51]	*n* = 265 (M: *n* = 96; F: *n* = 183)	44.9% 2-dimension criteria (EE + CY) 26.4% 3-dimension criteria (EE + CY + LOW AE)	MBI-SS	EE: 70.6%; CY: 52.8%; LOW AE: 48.7%.	First year: poor self-perception of health, not being optimistic, not feeling fulfilled as a medical student, long time spent in college, feeling worn out and dissatisfied; demotivation to study, low frequency of family meetings, lack of leisure time, low physical activity.	Good self-perception of health, optimism, motivation to study, non-exhaustive study routine.
21	Colby et al., South Africa (2018) [52]	*n* = 91 (Fourth Year)	46.1%	MBI WHOQOL-BREF	EE: *n* = 36; 39.6%; D: *n* = 38, 41.7%; PA: *n* = 53; 58.2%.	Health subscale of the WHOQOL-BREF associated with all three subscales of the MBI, in particular EE.	
22	Erschens et al., Germany (2018) [53]	*n* = 597 (M: *N* = 225; F: *N* = 372)	35%	MBI-SS	High EE: 23.5%; High CY: 17.8%; Low AE: 35.8%.	Students in earlier stages showed higher values for EE.	Support from families, friends, and students; relaxing exercise; sport.
23	Liu et al., China (2018) [54]	*n* = 453 (M: *n* = 199; F: *n* = 254)	9.27% M: 11.56% F: 7.48%	MBI-SS	EE: 3.42 (±1.45); CY: 2.34 (±1.64); LOW PE: 3.04 (±1.30).	Upper grades associated with higher CY and lower PE.	
24	McLuckie et al., Canada (2018) [55]	*n* = 232	23.3%	MBI-2	EE: 22.3 ± 14.7; D: 8.5 ± 9.1.	Higher years and concern about mental health were associated with higher EE.	
25	Talih et al., Lebanon (2018) [56]	*n* = 172 (M = 88; F = 84)	43%	BMS	Not available	Male gender; not living with parents; stressful life events in the past year; sleeping less than 6 h/day; current suicidal ideation; caffeine consumption; self-prescribed psychotropics; fourth-year medical student.	
26	Al-Alawi et al., Oman (2019) [57]	*n* = 662 (M:* n* = 27.6%; F: *n* = 72.4%)	7.4%	MBI-SS	High EE: OR 3.52; High CY: OR3.33; Low PE: OR 2.07.	Being 21 years old or younger, being preclinical students.	
27	Asghar et al., Pakistan (2019) [58]	*n* = 600 (M: *n* = 203; F: *n* = 397)	18.2%	MBI	EE female: low 98 (16.3%); moderate 175 (29.2%); high 124 (20.7%). EE male: low 64 (10.7%); moderate 101 (16.8%); high 38 (6.3%). D female: low 120 (20%); moderate 170 (28.3%); high 107 (17.8%). D male: low 50 (8.4%); moderate 89 (14.8%); high 64 (10.7%). PA female: high 99 (16.5%); moderate 179 (29.8%); low 119 (19.8%). PA male: high 56 (9.3%); moderate 102 (17.1%); low 45 (7.5%).	Sleeping less than six hours; not sharing concerns and problems with anyone; incapacity of controlling anger; having no hobbies due to time constraints; or not having mental energy for recreational pursuits.	
28	Calcides et al., Brazil (2019) [59]	*n* = 184 (M: 54.9%)	10.3% (3-dimension criteria) 35.9% (EE + CY)	MBI-SS	High EE: 53.3%; High CY: 52.2%; Low PE: 19.0%.	Thoughts of quitting program, unsatisfactory perception of academic performance and teaching strategies, use of licit psychoactive substance.	Participation in Balint Group.
29	Cortez, et al., USA (2019) [88]	*n* = 62 (Pre- and post-surgical clerkship)	Before surgery clerkship: 22.6% After surgery clerkship: 17.7%	12-item Grit Scale MBI	Before surgery clerkship: EE: 22 (14–17); D: 7 (3–11); PA: 36 (32–39). After surgery clerkship: EE: 21 (16–29); D: 8 (3–12); PA: 38 (32–40).	Lower Grit score. No increase in medical student burnout following the surgery clerkship.	Increasing Grit score (decreasing EE and D, increasing PA)
30	Fitzpatrick et al., Ireland (2019) [60]	*n* = 268	35% in clinical years; 26% in preclinical years	MBI-SS	High EE: OR 3.52; High CY: OR 3.33; Low PE: OR 2.07.		
31	Haile et al., Ethiopia, (2019) [61]	*n* = 144 (M: *n* = 123; F: *n* = 21)	34%	MBI-HSS	High EE: 61.8%; High D: 47.9%; Low PA: 59.7%.	Being less than satisfied by practice lecturers, poor social support, being less than satisfied by the education system.	
32	Isaac et al., Australia (2019) [62]	*n* = 638 medical students during rural clinical placement (M: *n* = 265; F: *n* = 373)	26.5%	Single-item validated assessment	Not available	Female gender, rural origin, previous stress level, isolation during rural placement.	
33	Ofei-Dodoo et al., USA (2019) [63]	*n* = 379	48%	MBI	EE: 42.7%; D: 26.5%.	High EE, high D, manifestations of burnout, increased with year in training.	
34	Puranitee et al., multisite (2019) [64]	*N* = 451 (M: *n* = 225; F: *n* = 226)	28.4%	MBI-SS	LOW PA: 54.8%; EE: 57.4%; D: 65%.	Male gender, lower academic performance.	
35	Wilkes et al., Canada (2019) [65]	*n* = 69 (F: *n* = 75%; M: *n* = 22%)	64%	OLBI, CAGE, GHQ-12	DI: 64%; EX: 70%.		
36	Alkhamees et al., Saudi Arabia (2020) [66]	*n* = 305 (M: *n* = 144; F: *n* = 161)	5.6%	MBI-SS	Not available	Female gender, being in clinical class; when the adjusted OR is considered, premed students were more likely than their peers in clinical to develop burnout	Age between 18–21 years, male gender, being in premed class.
37	Armstrong et al., USA (2020) [67]	*n* = 138	PB: 50%	CBI	Study-related burnout: 42%; Client-related burnout: 12%.	Being women for work-related burnout and PB; being black for PB.	
38	Guang et al., USA (2020) [31]	*n* = 51 (M: *n* = 16; F: *n* = 35)	31.4%	MBI	Not available		Gap years before medical school.
39	Kajjimu et al., Uganda (2020) [68]	*n* = 145 (M: *n* = 102; F: *n* = 43)	54.5%	MBI-SS	EE: 93.1%; CY: 97.2%; LOW PE: 62.1%.	Choosing Bachelor’s degree in Medicine and Surgery.	
40	Lee et al., Hong-Kong (2020) [69]	*n* = 731 (M = 44.2%; F = 55.8%)	27.9%	MBI	EE: 49.3% (95% CI: 45.5–53%); D: 53.8% (95% CI: 49.9–57.5%); Low PA: 71.1% (95% CI: 67.5–74.4%).	Using alcohol more than 4–5 times per week; medical degree as their first degree, to live in the hospital dormitory, higher PSQI scores.	Performing regular exercise.
41	Morgan et al., Canada (2020) [70]	*n* = 129 (M: 42.6%; F = 56.6%; Other: 0.8%)	20.9%	2-item MBI	Not available	Being a woman; being in third or fourth year.	
42	Nteveros et al., Cyprus (2020) [71]	*n* = 189 (M = 68; F = 121)	18.1%	MBI-SS	Not available	Increasing academic year, especially after the 4th. Poor sleep quality and worse mental health, alcohol consumption (CY).	
43	Mahfouz et al., South Arabia (2020) [72]	*n* = 438 (M: *n* = 205; F: *n* = 233)	60.2% 64.1% F 56.2% M	CBI	PB: 20.3%; SB: 18.6%; CB: 23.6%.	Poor sleep.	Adequate sleep, physical activity, psychological support, educational strategies, better learning environment.
44	Obregon et al., USA (2020) [73]	*n* = 273 (M: *n* = 112; F: *n* = 158)	40.3%	MBI-SS	EE: 23.23 ± 4.74; CY: 14.44 ± 5.59; Low AE: 24.81 ± 5.35.	Female gender, out-of-phase.	Motivation.
45	Perni et al., USA (2020) [74]	*n* = 209 (M: 41%; F: 59%)	39%	2-item MBI	Not available	Students in the highest tertile of composite moral distress scores were more likely to be burnt-out (51%) than those in the middle tertile of scores (34%), or lowest tertile of scores (31%) (*p* = 0.02).	
46	Tavares et al., Brazil (2020) [75]	*n* = 419 (F: 64.7%)	9.5%	MBI-SS	EE: 38.4; D: 29.4; LOW PE: 32.7.	EE: use of stimulating substances. DP-LOW PE: the use of tobacco; alcohol. EE-PD-LOW PE: thinking about giving up.	More hours of sleep; better stress management.
47	Aghajani Liasi et al., Iran (2021) [76]	*n* = 123	16.3% F = 96 (80.7%) M = 23 (19.3%)	MBI	Normal *n* = 60 (62.5%); Depression *n* = 36 (37.5%); Anxiety *n* = 39 (41.1%); Stress *n* = 30 (30.1%).		
48	Aljadani et. al., Saudi Arabia (2021) [77]	*n* = 218 (F = 121; M = 97)	27.1%	MBI	EE: 79.4%; CY: 61.0%; PE: 37.6%.	EE: female gender; final year of medical school.	High grade point average.
49	Alqifari et al., Saudi Arabia (2021) [78]	*n* = 336 (M: 56.5%; F: 43.5%)	8%	MBI-SS	EE: 29.5%; CY: 33.3%.	EE-PE: female gender.	
50	Dias et al., Brazil (2021) [79]	*n* = 209 (F: 57.9%)	28.2%	MBI-SS	Not available	Depressive symptoms.	Resilience, being older, being married, or having better academic performance.
51	Ilic et al., Serbia (2021) [80]	*n* = 760 (M: *n* = 269; F: *n* = 491)	15% (M: 19%; F: 12.8%)	MBI-SS	Not available	F: use of sedatives; cigarette smoking; F-M: third academic year; alcohol consumption.	
52	Prata et al., Brazil (2021) [81]	*n* = 213 (M: 50.2%; F: 49.8%)	21.6%	MBI-SS	EE and CY: 51.6%. EE: Low: 11.3%; Moderate: 17.4%; High: 71.4%. CY: Low: 10.3%; Moderate: 32.4%; High: 57.3%. PE: Low: 25.8%; Moderate: 18.3%; High: 55.9%.	Rarely receiving the emotional support needed during the program; thinking about dropping out of the undergraduate; considering one’s academic performance to be regular or weak.	
53	Samuels et al., USA (2021) [32]	*n* = 26,123 (M: *n* = 13,470; F: *n* = 12,653; LGB: *n* = 1410)	Heterosexual medical students: 11.1% LGB medical students: 17.2%	OLBI	Heterosexual medical students: Disengagement: 21.01%; Exhaustion: 22.5%. LGB medical students: Disengagement: 27.8%; Exhaustion: 30.6%.	Sexual minority, regardless of perceived experience of mistreatment.	
54	Thun-Hohenstein et al., Austria (2021) [89]	*n* = 135 (M: 42.2%; F: 57.8%)	T1: 33.9% T2: 60.9% T3: 48.5% COMBINED: 49%	MBI Six Factors Theory of Burnout	EE T1: 3.97 (0.90); T2: 4.55 (0.87); T3: 4.25 (1.02); CY T1: 2.51 (0.91); T2: 3.11 (1.26); T3: 2.88 (1.21); LOW PE T1: 2.10 (0.63); T2: 2.46 (0.72); T3: 2.36 (0.70). High workload, high external control, low reward, low feeling of community, low fairness.	Increasing from youngest to oldest class; academic term (T2 > T3 > T1).	
55	Yahya et al., Iraq (2021) [82]	*n* = 424	38.2%	MBI-SS	EE: 85.6%; CY: 77.8%; Low PE: 32.8%.	Female gender, regular use of legal substances, and family history of mental diseases.	
56	Ahmadabadi et al., Iran (2022) [83]	*n* = 668 (M = 332; F = 336)	24.1%	MBI-SS	EE: 65%; CY: 75.4%; Low AE: 37%.		
57	Bolatov et al., Kazakhistan (2022) [84]	*n* = 736 (M = 184; F = 552)	CBI-S 28% OLBI-S 31%	CBI-S OLBI-S	PB: 58.8%; SB: 54.8%; COB: 13.0%; TB: 25.4%; EX: 16.2%; D: 23.5%.		
58	El-Gabry et al., Egypt (2022) [85]	*n* = 547 (M = 261; F = 286)	88%	OLBI	Total DI score: 20.48; Total EX score: 22.14; Mean DI score: 2.56; Mean EX score: 2.77.	Positive correlation between the OLBI and the GHQ12.	
59	Nassar et al., Brazil (2022) [86]	*n* = 94 (M: *n* = 42; F: *n* = 52)	23.4%	MBI-SS	EE: 57%; CY: 66%; Low PA: 37%.		
60	Qashqary et al., Saudi Arabia (2022) [87]	*n* = 271 (M: 73%; F: 27%)	19.6%	MBI	High level burnout: 23.3%; High D: 70.5%; High PA: 79.3%.	Male gender, second year.	
61	Gilbey et al., Israel (2023) [27]	*n* = 966	50.6%	MBI-SS	EE: 70.4%; CY: 57.1%.	Female gender, younger age, advanced year of study, attending a specific medical school, and not being a parent.	
62	Mhata et al., South Africa (2023) [28]	*n* = 229 (M: 28.4%; F: 71.6%)	36.2%	MBI-SS	EX: 68.1% (*n* = 156); CY: 77.3% (*n* = 177); EF: 53.3% (*n* = 122).	EX and CY are related with female gender.	
63	Thew et al., Malaysia (2023) [29]	*n* = 328 (M: *n* = 113; F: *n* = 215)	10.1%	MBI-SS	Engaged: 25.9%; Ineffective: 57.6%; Overextended: 31.7%; Disengaged: 11.9%.	Number of collected merits, smartphone addiction, hours spent on the smartphone most days in the last month, family problems, family pressure, poor communication with friends and lecturers, high self-expectation in examinations, too many examinations.	
64	Zaidi et al., Pakistan (2023) [30]	*n* = 284	52.5%	OLBI		Higher academic year more than 2 times and living at dormitory more than 3 times were associated with more extensive overlap of burnout and depressive symptoms.	

AE: academic efficacy; BMS: Burnout Measure—Short version; CAGE: Cut-down–Annoyed–Guilty–Eye-opener for alcoholism; CB: Client-related burnout; CBI: Copenhagen Burnout Inventory; CBI-S: Copenhagen Burnout Inventory—Student Survey, GHQ-12: General Health Questionnaire—12 items; COB: colleague-related Burnout; CY: cynicism; D: depersonalization; DI: disengagement; EE: emotional exhaustion; EX: exhaustion; GWB: General Well-Being Schedule; K-10: Psychological Distress Scale; MBI: Maslach Burnout Inventory; MBI-GS: Maslach Burnout Inventory—General Survey; MBI-HSS: Maslach Burnout Inventory—Human Services Survey; MBI-SS: Maslach Burnout Inventory—Student Survey; MBI-2: Maslach Burnout Inventory—2 items; OLBI: Oldenburg Burnout Inventory; OLBI-S: Oldenburg Burnout Inventory for college students; PA: personal accomplishment; PB: personal burnout; PE: professional efficacy; PMSSS: Perceived Medical School Stress Scale; PRIME-MD: Primary Care Evaluation of Mental Disorders; PS: persistence; QOL: Quality of Life Scale; SB: Study-related burnout; SF-8: Health-Related Quality of Life Short Form 8; ST: Self-transcendence; TB: Teacher-related burnout; WHOQOL-BREF: World Health Organization Quality of Life—Brief version.

### 3.1. Assessment Tools

Burnout was assessed by means of the Maslach Burnout Inventory and its related versions (MBI—General Survey; MBI—Human Services Survey; MBI—Student Survey, 2-item MBI), the Oldenburg Burnout Inventory [30,32,49,65,84,85], the Copenhagen Burnout Inventory [67,72,84], and the Burnout Measure—Short form [56]. Isaac et al. [62] explored the presence of burnout in medical students during a rural placement in Australia by using a validated single-item assessment. In most studies, burnout was assessed alongside distress, stress, quality of life, and general well-being using validated assessment tools, such as the Kessler Psychological Distress Scale [90], the Perceived Medical School Stress Scale [91], the Health-Related Quality of Life Short Form 8 [92], the Quality of Life Scale [93], the World Health Organization Quality of Life Brief version [94], and the General Well-Being Schedule [95].

### 3.2. Dimensions of Burnout

Among the dimensions of MBI, Kajjimu et al. [68] reported the largest prevalence of high levels of emotional exhaustion (93.1%) and of cynicism (97.2%), while Qashqary and colleagues [87] detected high levels of depersonalization in more than 70% of medical students; low personal accomplishment was the most frequently reported burnout dimension in the study by Lee et al. [69].

### 3.3. Prevalence Rates

The prevalence rate of burnout ranged from 5.6% [66] to 88% [85]. In some studies, the prevalence rates were stratified according to gender, with a burnout rate of 80.7% in female students [76] and 56.2% in males [72]. Female students reported higher levels of emotional exhaustion (20.7% vs. 6.3%) and of depersonalization (17.8% vs. 10.7%), and lower levels of personal accomplishment (7.5% vs. 19.8%) [58]. The female gender was a significant predictor of burnout in most studies [27,28,42,49,50,62,66,67,70,73,77,78,82], with the exception of the studies by Cecil [38], Talih [56], Puranitee [64], and Qashqary [87].

After stratifying for the stage of the medical course, increasing rates of burnout from the preclinical years (2.8% and 26%) to clinical years (8.3% and 35%) were found by Asencio-López et al. [44] and Fitzpatrick et al. [60], respectively. Cortez et al. [88] detected burnout in 22.6% of students before attending their surgery clerkship, and in 17.7% of them afterwards; Thun-Hohenstein et al. [89] found a rate of burnout of 33.9% at the beginning of the academic year (T1), 60.9% at the end (T2), and 48.5% at the beginning of the following one (T3).

### 3.4. Predictors of Burnout

The stages of medical education were associated with overall levels of burnout, being higher in students attending advanced years [27,30,46,54,55,56,63,70,71,77,89]. Factors predicting high levels of burnout were thoughts of stopping medical education [43,59,75,81], negative or stressful life events [26,42,56], a lack of perceived support from family and peers [45,48,58,61,81], dissatisfaction and lack of motivation [40,51,59,61], being part of sexual minority [32] and the experience of recurrent mistreatment at university [48]. In fact, students receiving recurrent mistreatment by faculty members or residents reported burnout rates of 57.4% and 49.1%, respectively [39]; higher levels of disengagement (27.8%) and exhaustion (30.6%) were found among lesbian–gay–bisexual medical students when compared with heterosexual colleagues [32]. Moreover, poor sleep and the use of tobacco, caffeine, alcohol, and illicit substances were also associated with burnout [38,56,58,59,69,71,72,75,80,82].

### 3.5. Protective Factors

Factors protecting against burnout were social and emotional support, also provided by counselling services or Balint groups [37,42,59], and relaxation and physical exercise [46,53,69,72].

## 4. Discussion

Young people attending university courses face a crucial stage of personal development, with increasing pressure to acquire knowledge and skills [96,97,98]. Moreover, the academic context is characterized by peer pressure and competition, limited socioeconomic power, and distance from home and family, which represents a significant stressful factor for students. Therefore, academic burnout has become a relevant factor affecting college students’ mental health. Although high levels of burnout are common among university students, medical students are particularly vulnerable because of the persistent exposure to specific risk factors, such as the caring of people suffering from different disorders, and the high competitiveness in medical school [99,100]. In particular, the tough competition among medical students could probably be considered as a motivational drive that is helpful in overcoming the several obstacles of the training curriculum. Medical students’ competition may result in the pressure to succeed in school performance, in learning to stay on top of the classmates, and in limited time for leisure and relaxation, which may commonly contribute to the generation of high levels of stress.

The levels of burnout in medical students may reach alarming peaks [85], requiring greater attention and the implementation of support services aimed at preventing further negative mental health outcomes. Based on such premises, the present systematic review has been carried out for investigating the prevalence rates, assessment tools, and possible predictive factors of burnout in medical students.

According to the present review, the prevalence rate of burnout ranged from 5.6% [66] to 88% [85], which should be partly explained by the different assessment tools adopted, in particular, the findings by Alkhamees et al. [66], reporting a rate of 5.5% by using the student survey of MBI, and those by El-Gabry et al. [85], who detected a proportion of 88% by means of the OLBI. Burnout symptoms are usually evaluated with different assessment tools, whose accuracy and reliability have been proven [101]. The Maslach Burnout Inventory (MBI) was originally developed for assessing emotional exhaustion, depersonalization, and low personal accomplishment in human service and healthcare professionals [102], while special versions of this instrument were validated for educators [102], workers in general [103], students [19], and medical staff [104]. Several versions of the Maslach Burnout Inventory have been developed to measure the subtle burnout differences among different target groups. Further assessment tools not based on the three-dimension conceptualization of burnout, such as the Burnout Measure scale [105], the Oldenburg Burnout Inventory (OLBI) [106], the Copenhagen Burnout Inventory [107], the Shirom Melamed Burnout Measure [108], and the Burnout Assessment Tool [109], have been more recently proposed. As our findings show, the Maslach Burnout Inventory is the most used tool to assess burnout levels. However, the availability of different instruments and several versions for the same instrument should have contributed to the extreme variation in prevalence rates of burnout worldwide. Therefore, it would be useful to identify the best assessment for the evaluation of burnout in medical students. This finding is also confirmed by the high rate of burnout found in healthcare professionals, with more than 40% of physicians reporting burnout at any given time [110].

One of the aims of the present review is to identify predictors and protective factors for burnout syndrome in medical students. We found that the female gender is significantly associated with a higher risk of burnout syndrome compared to the male gender [27,28,42,62,66,67,70,73,77,78,82]. This should be due to the fact that female medical students may experience higher vulnerability to distressing conditions [28,82], further demands outside the university context [28], and the difficulties in balancing their personal and professional life [27]. On the other hand, four studies found higher levels of burnout among male students compared to female ones [38,56,64,87], reflecting the general inconsistency of the relationship between gender and burnout in terms of strength and direction [111]. A meta-analysis found that women are slightly more emotionally exhausted than men, while men are somewhat more depersonalized than women [112]. These data, if confirmed, highlight the need to implement and disseminate personalized and gender-based approaches for the prevention of burnout in medical students.

Further risk factors commonly reported in association with burnout syndrome include negative life events [26,42,56], a lack of perceived support [45,48,58,61,81], a sense of dissatisfaction and lack of motivation [40,51,59,61], belonging to a sexual minority [32], recurrent mistreatment at university [39], logistic difficulties [62], and a poor lifestyle, including the misuse of substances [38,56,58,59,69,71,72,75,80,82]. Moreover, medical students have to face an intrinsically stressful curriculum, which places significant demands on them [113], such as passing a large number of theoretical and practical examinations, requiring a heavy workload during the preparation phase [29]. Furthermore, these subjects have to be versatile in switching between topics that may differ a lot from one another, regardless of the medical specialty they intend to pursue. Indeed, surgical branches require the application of different skills and attitudes when compared with basic sciences (e.g., genetics and biology), internal medicine, pharmacology, or clinical specialties such as pediatrics or psychiatry. Moreover, many medical specialties are characterized by the inherent complexity of learning and demand massive mnemonic efforts. The physiological and pathological processes of the human body, as well as therapeutic procedures, are required to be known from the molecular to macroscopic levels.

Difficulties may vary according to the stage of the study career. In particular, some medical students experience problems during the transition from school to the university, that may also be explained by considering the efforts and the long time needed to pass the admission exam [51]. Higher levels of burnout [51,57,87] and exhaustion [53] have been reported during the early stages of medical school, characterized by the adaptation to new difficulties and challenges [51,87]. However, several studies [27,30,44,46,54,55,56,60,63,70,71,77,89] showed that the clinical years seemed to be more critical for mental health. In fact, alongside many commitments (e.g., attending lectures, preparing assignments, completing lab work, and choosing a medical specialty), medical students attending the last years are also required to learn how to manage patients’ demands and build therapeutic relationships in practice. Moreover, they have to spend rotations periods, with a continuous need for adaptation, which impacts their mental health. During the advanced stages of medical school, students are exposed to highly distressing situations [114], such as sickness and death, which represent further reasons of vulnerability.

Gilbey et al. [27], who surveyed medical students mostly aged 26 or older, found that the highest levels of burnout were associated with the youngest age. It is relevant to consider that the age of these subjects (usually ranging between 19 and 30 years) falls into a critical period for the onset of several mental disorders including psychosis, and affective and anxiety disorders [21]. According to a large meta-analysis including more than 700,000 individuals, 62.5% of the sample presented the onset of any mental disorder before the age of 25 [115]. Such findings suggest the importance of focusing on this period of life to detect any anomalies and take early action. Moreover, overcoming the separation of child/adolescent and adult services, which still characterizes the mental healthcare system, should represent the way forward for mental health systems. The implementation of new preventive strategies, the development of training programs, and the involvement of “allied” professionals, such as primary care or emergency doctors and school staff, have also been evoked for optimizing the assistance of mental health in youth [116]. Recently, several international scientific associations, such as the World Psychiatric Association (WPA), have established a specific working group dedicated to medical students. In particular, this working group aims to identify various areas of promoting psychiatry as a career among medical students and to support medical students around the world.

Given the heterogeneity of the identified risk factors, it is out of the scope of the present review to identify the most relevant risk factor. However, it should be highlighted that medical students can be exposed to several risk factors acting at the same time, with a complex interplay among different factors, further improving the levels of burnout.

Once it is developed, burnout is a self-sustaining process, if not adequately treated. Specific and tailored supportive interventions for medical students and early career physicians should be developed, since they are exposed to a disproportionate risk of mental health problems leading to suicidal ideation. Treatments, including pharmacotherapy, psychotherapy, or their combination, have been proven to be effective in managing clinical symptoms due to burnout syndrome [117].

Moreover, since the levels of stigmatizing attitudes and behaviors toward their mental health are higher in medical students compared to other peers of the same age, anti-stigma campaigns targeting medical students should be carried out [118,119,120,121]. This finding is probably due to the fact that stereotypes about mental health and mental disorders are not adequately addressed during medical training, and students may feel discriminated against in disclosing their mental health problems, with delays in seeking help and access to mental health care. In fact, some studies found that medical students agree that clinicians cannot have any mental disorders and are concerned about being considered unfit to practise as physicians if they suffer from mental health problems [122]. This finding is in line with the low access rate of medical students to psychological and psychiatric support available in many universities due to the perceived and internalized stigma, lack of knowledge about existing services, and poor confidence towards available mental health treatments.

Based on the present systematic review, we found that social and emotional support, attending counselling services or Balint groups, practicing relaxation, and performing regular physical exercise were protective against the risk of burnout. These latter findings are in line with the vision of promoting the mental health of young people by encouraging them to pursue a healthy lifestyle, in order to reach higher levels of general wellbeing [1]. The implementation of such programs and facilities within medical schools could contribute toward decreasing burnout levels.

Some limitations of our review should be acknowledged. Firstly, although the reviewed studies were conducted in different regions of the world, low- and middle-income countries were poorly represented. Furthermore, the extreme heterogeneity of the included countries should be considered when interpreting the results, since differences exist in terms of national academic curricula, including the number of exams, the duration of training, practical activities, theteachers’ availability, logistic facilities, and financial concerns. Another limitation is the adoption of self-reported questionnaires, without a confirmation of the mental health status by a clinical psychiatric examination. Finally, we decided to exclude all studies carried out during the COVID-19 pandemic, since this may have represented a trigger and stressful factor per se which has changed everyone’s daily routine, particularly that of young people and of students. In fact, alongside the global distress due to fear of contagion, grief, restraint measures, the lack of social relationships, and limited leisure activities, many medical students experienced distance learning. This way to attend academic class allowed the continuation of lessons in a safe way, but it required abrupt adaptation, having important implications in the development of practical and behavioral skills, and on social contact with peers. Furthermore, a recent meta-analysis and systematic review by Peng et al. [123] found high rates of burnout, depression, anxiety, stress, insomnia, psychological distress, PTSD, and suicide ideation among medical students during the period of the COVID-19 pandemic [6,124].

## 5. Conclusions

The term “burnout” was first introduced in the psychological research in order to describe the sense of physical, emotional, and interpersonal exhaustion experienced by healthcare workers, traditionally recognized as a group at risk. Throughout the last decades, several instruments have been developed in order to evaluate the prevalence of burnout syndrome in different at-risk categories, including also medical students.

Students enter medical school have a mental health status very similar to their same-age peers, but, during medical school, their mental health severely deteriorates.

Medical students are the doctors of tomorrow. They are called upon to prepare themselves to face and manage suffering. Because of this, they may feel the burden of future responsibilities and of social expectations in this regard. Furthermore, they also have to deal with a stressful routine on a daily basis, in which lectures, practical activities, and preparation for a number of exams fit into the thick pages of their agendas, in which free time might not find much space. Despite this, burnout among medical students has not been investigated enough so far [24].

Students often develop comorbid mental health conditions as a consequence of burnout. Students experiencing burnout were approximately 3.5 times more likely to experience thoughts of self-harm, and it is associated with an increased risk for suicidal ideation.

Our findings confirm that burnout represents an epidemic in the population of medical students. Indeed, the prevalence rates may reach alarming peaks, despite the fact that the use of different assessment tools results in a wide heterogeneity. Therefore, preventive and supportive strategies should be implemented by medical universities to manage this public mental health problem [125,126].

International societies such as the WPA have dedicated a specific initiative in order to support medical students, by providing educational resources, teaching sessions, and online training programs [127,128,129].

## Data Availability

The data are available upon request from the corresponding author.

## Supplementary Materials

The following supporting information can be downloaded at: https://www.mdpi.com/article/10.3390/medicina60040575/s1, Table S1: Risk of bias assessment in included studies. Reference [130] are cited in the Supplementary Materials.
